# Characteristics and outcomes of secondary hematological malignancies following autologous stem cell transplantation for multiple myeloma

**DOI:** 10.1038/s41408-026-01537-4

**Published:** 2026-06-23

**Authors:** Binoy Yohannan, Benjamin W. Langworthy, Lindsey E. Turner, Angela Dispenzieri, Francis K. Buadi, David Dingli, Nelson Leung, Prashant Kapoor, Wilson I. Gonsalves, Taxiarchis Kourelis, Joselle Cook, Moritz Binder, Suzanne R. Hayman, Yi Lin, Ronald S. Go, Rahma M. Warsame, S.Vincent Rajkumar, Shaji Kumar, Eli Muchtar, Hassan B. Alkhateeb, William J. Hogan, Aasiya Matin, Mrinal M. Patnaik, Aref Al-Kali, Abhishek A. Mangaonkar, Hefazi Torghabeh M, Robert C. Wolf, Mithun V. Shah, Morie A. Gertz

**Affiliations:** 1 Division of Hematology, Mayo Clinic, Rochester, MN USA; 2 Division of Hematology, Oncology and Transplantation, University of Minnesota, Minneapolis, MN USA; 3 Division of Biostatistics and Health Data Science, School of Public Health, University of Minnesota, Minneapolis, USA; 4 Department of Pharmacy, Mayo Clinic, Rochester, MN USA

**Keywords:** Risk factors, Myeloma

## Abstract

Autologous stem cell transplantation (auto-SCT) remains an important pillar in multiple myeloma (MM) therapy; however, there is an increased risk of developing second primary malignancy including second hematological malignancy (SHM). We performed a retrospective study of patients with MM who underwent an auto-SCT at our institution between 1990 and 2022 and subsequently developed SHM. Among 3401 patients with MM who underwent auto-SCT, 110 (3.2%) developed SHM [therapy related myeloid neoplasm (t-MN) = 98; acute B cell lymphoblastic leukemia (t-B-ALL) = 11 and mixed phenotype acute leukemia=1). The cumulative incidence of SHM has increased between the pre-novel (auto-SCT, 1990–2006) and novel agent eras (2007- 2022) (1.1% vs 2.0% at 60 months; *P* = 0.03). Somatic mutations in *TP 53* gene were seen in 40% of patients with t-MN. Among t-B-ALL patients, high incidence of hypodiploidy (*n* = 4) and *IKZF1* (*n* = 3) deletion was observed. In multivariate analysis, receiving an alkylator based induction, radiation therapy, achieving a complete response after auto-SCT, use of cyclophosphamide for stem cell mobilization and lenalidomide maintenance were independent predictors of developing SHM. Patients with MM who developed a SHM had an inferior overall survival (OS). Given the median OS for MM continues to improve, monitoring for SHM and risk mitigation strategies is crucial.

## Introduction

Multiple myeloma (MM) is a clonal plasma cell disorder which accounts for approximately 10% of all hematological malignancies[1]. The clinical outcomes of patients with MM have improved dramatically in the era of novel therapies with a median overall survival (OS) of more than 10 years[2–4]; however, MM still remains incurable in most patients. High-dose melphalan followed by autologous stem cell transplantation (auto-SCT) remains an important pillar of MM therapy. However, there is an increased risk of second primary malignancies (SPM), including solid tumors and second hematological malignancies (SHM). In the Surveillance, Epidemiology, and End Results (SEER) database study of 60,550 MM patients [1992–2020], 6.07% developed SPM and among them 0.8% developed SHM[5].

Population based studies have shown that patients with MM and its precursor diseases have a significantly higher risk of developing myelodysplastic syndrome (MDS)/acute myeloid leukemia (AML)[6, 7]. In addition, therapy related factors such as exposure to alkylating agents and ionizing radiation clearly increases the risk of SPM/SHM[8, 9]. Furthermore, the role of post-transplant lenalidomide maintenance in SPM/ SHM is well established[10].

SPM/SHM adversely affects the long-term outcomes of patients with MM[11]. Given lack of extended follow up in pivotal phase III MM trials[12], understanding the long-term toxicities associated with auto-SCT is critical as patients are living longer. In this study, we evaluated the clinical characteristics and outcomes of MM patients who developed SHM after auto-SCT treated at our institution.

## Materials and methods

We performed a single center retrospective study of MM patients who received an auto-SCT from January 1, 1990, to December 31, 2022, and subsequently developed a SHM. Patient clinical characteristics, laboratory data, treatment details and outcomes were obtained from electronic medical records. This study was approved by the institutional review board at Mayo Clinic, Rochester, Minnesota.

MDS was risk stratified based on the Revised International Prognostic Scoring System (IPSS-R)[13] and molecular IPSS (IPSS -M) was used when next generation sequencing (NGS) data was available[14]. AML was risk stratified using the 2022 European Leukemia Network risk (ELN) classification[15].

The time to SHM was calculated from the time of auto-SCT to SHM diagnosis. Patients were censored if they did not develop a SHM at the time of last follow up. OS was calculated from the date of auto-SCT to death from any cause and patients who were alive at the last follow up were censored. OS analysis for patients who underwent an allogenic stem cell transplant (allo-SCT) for their SHM was calculated from the date of allo-SCT. Non-relapse mortality (NRM) was calculated from time of allo-SCT to death from any cause other than disease progression or relapse.

The primary objective of this study was to investigate the incidence of SHMs in patients undergoing auto-SCT for MM and evaluate the risk factors associated with SHM.

We excluded patients with clonal hematopoiesis of indeterminate potential (CHIP) or clonal cytopenia of unknown significance and patients who developed a SHM within 3 months of an auto-SCT to rule out a previously undiagnosed neoplasm. The study was performed in accordance with the provisions of the Declaration of Helsinki.

### Statistical analysis

Descriptive statistics were used to analyze patients’ characteristics. Categorical variables were reported using frequencies and percentages and continuous variables using the median and range. For censored time-to-event data, the difference between the groups was analyzed using the log-rank test. We estimated the cumulative incidence of SHM accounting for death as a competing risk. Median follow up was calculated using the reverse Kaplan–Meier method. The cause-specific Cox proportional hazard regression model was used to estimate the effect of covariates on the hazard of survival. Similarly, Fine–Gray proportional hazards model was used to estimate the effect of covariates on subdistribution hazard for SHM taking into account death as a competing event. In both cases we selected which covariates to include using backwards selection with BIC (Bayesian Information Criterion) as the criteria for model selection. The key difference between the cause-specific Cox model and the Fine-Gray model is that the cause-specific model treats death as censoring, while the Fine-Gray model treats it as a competing risk. All *P* values shown were from 2-sided tests, and the reported confidence intervals (CIs) refer to 95% boundaries. Statistical analysis was performed using Graph pad prism version 10.5 and R version 4.4.1.

## Results

A total of 3401 patients with MM who underwent first auto-SCT between 1990 and 2022 were included. Patient characteristics are summarized in Table 1.

**Table 1:** Characteristics of patients who developed second hematological malignancy after auto-SCT for MM.

Variables	SHM (*N* = 110)	Non-SHM (*n* = 3291)	*P* value
Age (in years) at MM diagnosis, median (range)	60 (40–74)	60 (23–78)	0.66
Age (in years) at first auto-SCT, median (range)	61 (40–75)	61 (24–79)	0.49
Male	68 (62%)	1977 (60%)	0.77
**Myeloma subtype**			0.08
IgG	71 (64%)	1841 (56%)	
IgA	25 (23%)	656 (20%)	
IgD	1 (1%)	45 (1%)	
Light chain myeloma	12 (11%)	629 (19%)	
Other	1 (1%)	120 (4%)	
**ISS stage**			**<0.001**
I	49 (45%)	753 (23%)	
II	26 (24%)	814 (25%)	
III	21 (19%)	638 (19%)	
Missing	14 (13%)	1086 (33%)	
**Induction therapy prior to auto-SCT**			
Anti CD 38 monoclonal antibody	5 (5%)	276 (8%)	0.21
Proteasome inhibitor	71 (65%)	2166 (66%)	0.84
IMIDs	67 (61%)	2318 (70%)	**0.03**
Alkylating agents	43(39%)	953 (29%)	**0.03**
Melphalan	3 (3%)	97 (3%)	
Cyclophosphamide	32 (29%)	733 (22%)	
Melphalan and cyclophosphamide	8 (7%)	123 (4%)	
Topoisomerase II inhibitors	18 (16%)	408 (12%)	0.24
**Patients who received radiation therapy in any line of treatment**	36 (33%)	795(24%)	**0.04**
**Myeloma status at the time of first auto-SCT**			
PR or better	92 (84%)	2580 (78%)	0.37
MR/SD/PD	18 (16%)	705 (21%)	
Missing data	0	6 (<1%)	
**Time in months from diagnosis of MM to auto-SCT, median (range)**	6 (2–69)	6 (0–247)	0.71
**Stem cell mobilizing agent**			
Cyclophosphamide	25 (23%)	691 (21%)	0.64
Plerixafor	49 (45%)	1534 (47%)	0.70
**Conditioning therapy for first Auto-SCT**			0.76
Melphalan	108 (98%)	3076 (93%)	
Melphalan plus TBI	2 (2%)	110 (3%)	
Melphalan, cyclophosphamide and TBI	0	9 (<1%)	
BEAM	0	15(<1%)	
Proteosome inhibitor plus melphalan	0	56 (2%)	
Others	0	25 (<1%)	
**Total number of auto-SCT**			0.55
1	95 (86%)	2907 (88%)	
>1	15 (14%)	384 (12%)	
**Year of auto-SCT**			0.30
1990- 2006	30 (27%)	755 (23%)	
2007 -2022	80 (73%)	2536 (77%)	
**CR/sCR after auto-SCT**	55 (50%)	1113 (34%)	**<0.001**
**Lenalidomide maintenance**	43 (39%)	975 (30%)	**0.04**
**Duration of lenalidomide maintenance after auto-SCT**			0.18
<2 years	18 (42%)	310 (32%)	
>2 years	25 (58%)	665 (68%)	
**CAR-T cell therapy**	9 (9%)	161 (5%)	0.12
**follow-up time (months), median (range)**	269.6 (0.3–269.6)	106 (0–301.8)	**<0.001**

Bold *P* < 0.05. Other: includes non-secretory, IgE, IgM and biclonal.

*SHM* second hematological malignancy, *IMIDs* immunomodulatory drugs, *PR* partial response, *MR* minimal response, *SD* stable disease, *PD* progressive disease, *CR* complete response, *sCR* stringent CR, *ISS* international scoring system.

**Fig. 1:**
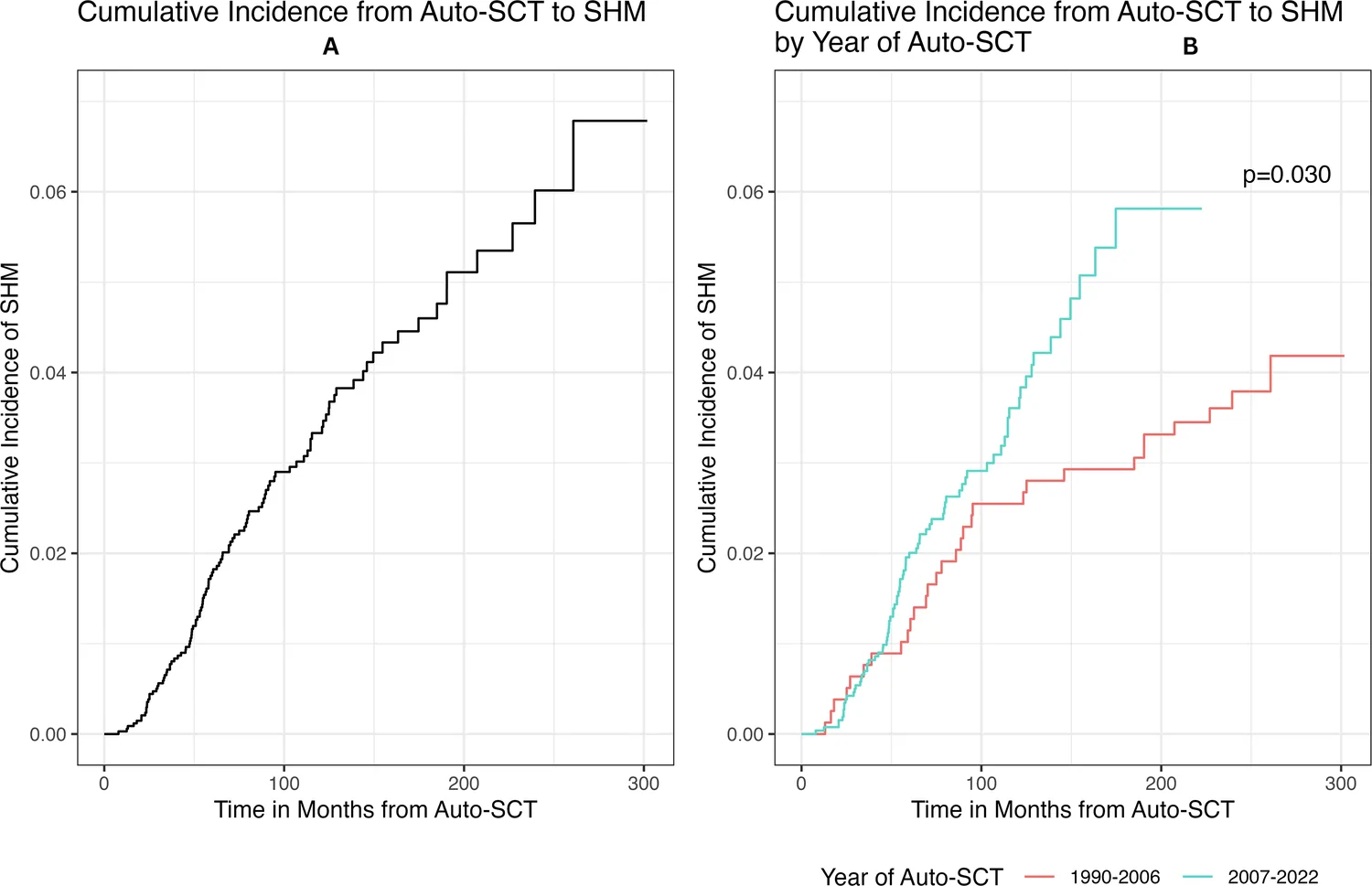
Cumulative incidence rate of developing second hematological malignancies accounting for death as a competing risk. **A** For the entire cohort: 1.8% (95%, 1.4–2.3%) at 5 years, and 3.4% (95% CI, 2.7–4.2%) at 10 years. **B** According to the years of transplant: auto-SCT (1990–2006) vs (2007–2022): 1.1% (95% C.I, 0.57–2.1%) vs 2.0% (95% C.I, 1.5–2.6%) at 60 months (*P* = 0.03).

At a median follow-up of 107.6 (range, 0.2–301) months from the date of auto-SCT, 110 (3.2%) patients developed SHM and among them, the majority (*n* = 98) were therapy related myeloid neoplasm (t-MN). Among CAR-T cell therapy recipients (*n* = 170), 9 patients (5%) developed t-MN. The incidence of SHMs were similar between patients who received idecabtagene vicleucel and ciltacabtagene autoleucel (8.2% vs3.2%, *p* = 0.18). The majority (n = 72) of SHMs occurred after the first 50 months from auto-SCT. A higher proportion of patients in the SHM cohort had stage I ISS, received alkylator based induction therapy, received lenalidomide maintenance therapy, achieved a complete remission (CR) after auto-SCT and received radiation therapy when compared to those without SHM (Table 1). The cumulative incidence of SHM accounting for death as a competing risk was 0.35% (95% CI, 0.20–0.61%)) at 2 years, 1.8% (95%, 1.4–2.3%) at 5 years, and 3.4% (95% CI, 2.7–4.2%) at 10 years (Fig. 1A) from auto-SCT. Further, the cumulative incidence of SHM has increased between the pre-novel [auto-SCT (1990–2006) 1.1% (95% C.I, 0.57–2.1%)] and novel agent era [2007–2022, 2.0% (95% C.I, 1.5–2.6%)] at 60 months (*P* = 0.03). (Fig. 1B). There was no significant difference in the time to SHM between patients who received auto-SCT from 1990–2006 compared to 2007–2022 (median of 81.9 months vs 54.8 months, *P* = 0.97).

**Fig. 2:**
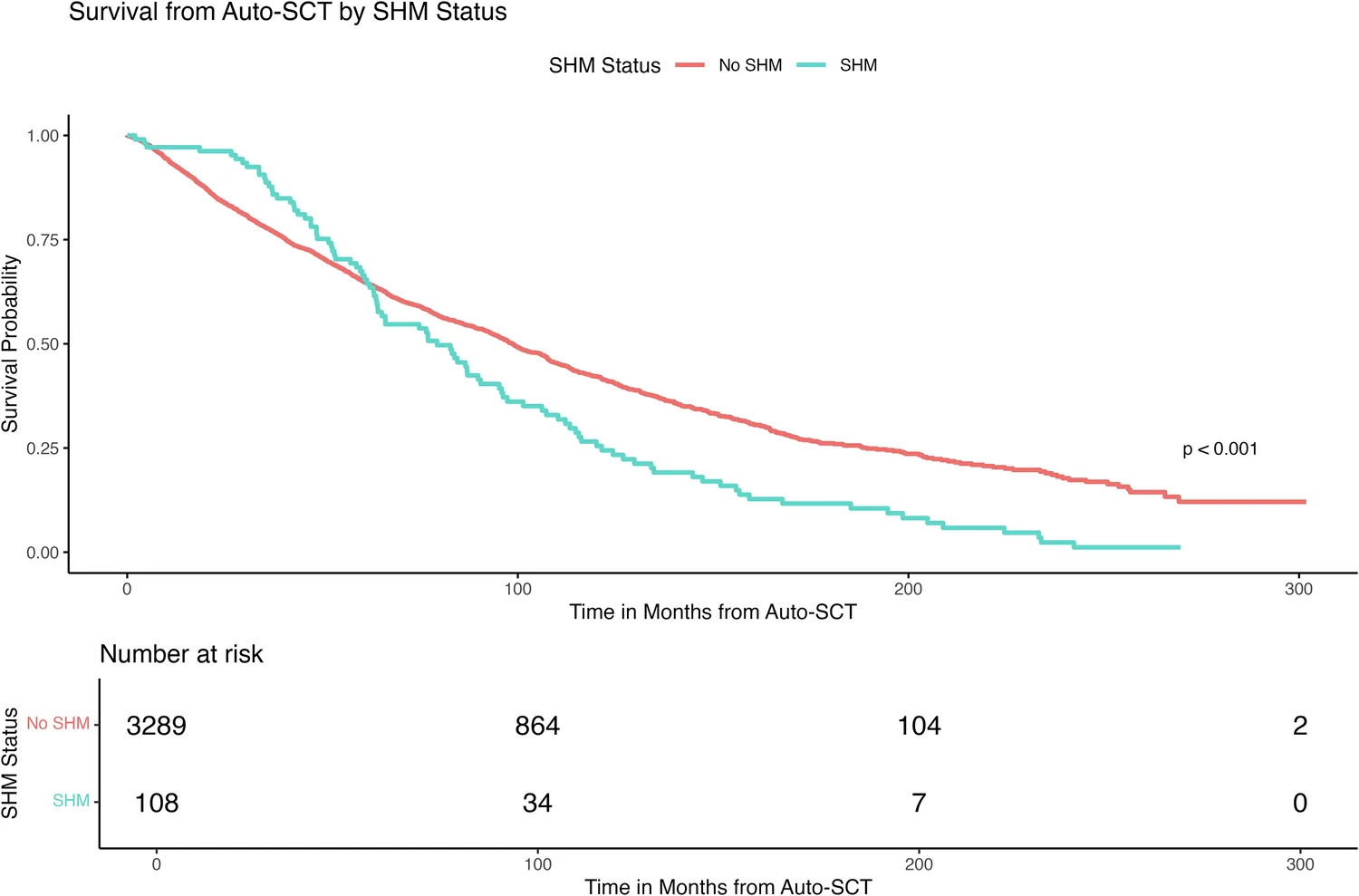
Overall survival in MM patients by second hematological malignancy status: median 81.3 months vs 98.7 months; hazard ratio 1.37, 95% C.I, 1.08–1.74; *P* = 0.001.

In a univariate analysis, induction therapy with an alkylating agent, radiation therapy, achieving a CR after auto-SCT and lenalidomide maintenance therapy were associated with increased risk of developing a SHM. In a multivariate regression analysis, in addition to the above-mentioned factors, use of cyclophosphamide for stem cell mobilization emerged as a predictive factor for SHM (Table 2A). According to the Fine-Gray model, receiving an alkylator based induction, radiation therapy, lenalidomide maintenance and achieving a CR after auto-SCT were independent predictors for developing SHM while accounting for death as a competing event (Table 2B). Patients with MM who developed a SHM after auto-SCT had an inferior OS when compared to those without SHM (81.3 months vs 98.7 months; hazard ratio 1.37, 95% C.I, 1.08–1.74; *P* = 0.001) (Fig. 2)

**Table 2:** A: Univariate and multivariate analysis of risk factors associated with second hematologic malignancy: Cox regression model. B: Fine-Gray Model.

A				
Variables	Univariate		Multivariate	
	Hazard ratio (95% CI)	*P* value	HR (95% CI)	*P* value
Age ≥ 60 at MM diagnosis	1.25 (0.86–1.83)	0.24	1.41 (0.95–2.10)	0.09
Gender (male)	1.32 (0.90–1.95)	0.16	Not included	
Induction therapy with alkylators	1.80 (1.23–2.65)	**0.003**	2.13 (1.44–3.16)	**<0.001**
Induction therapy with IMIDs	0.78 (0.53–1.15)	0.21	Not included	
Induction therapy with topoisomerase II inhibitors	1.56 (0.93–2.6)	0.09	Not included	
Year of auto-SCT				
1990- 2006	Reference	0.97	Not included	
2007 -2022	1.01 (0.64–1.59)			
> 1 Auto-SCT	0.73 (0.42–1.26)	0.25	Not included	
Conditioning regimen:			Not included	
Melphalan	Reference	0.47		
Melphalan + TBI	0.59 (0.14–2.43)			
Use of cyclophosphamide for stem cell mobilization	1.28 (0.82–2.02)	0.28	1.79 (1.11–2.91)	**0.02**
Use of plerixafor for stem cell mobilization	1.26 (0.85–1.88)	0.26	Not included	
Radiation therapy in any line of therapy	1.73 (1.16–2.57)	**0.007**	1.61(1.02–2.54)	**0.04**
CAR-T cell therapy in any line of therapy	1.19 (0.60–2.35)	0.62	Not included	
Lenalidomide maintenance post -auto- SCT	2.01 (1.34–3.00)	**<0.001**	2.59 (1.70–3.96)	**<0.001**
≥ CR after auto-SCT	1.58 (1.09–2.31)	**0.02**	1.68 (1.14–2.47	**0.01**

B		
Variable	Subdistribution Hazard Ratio (SHR) (95% CI)	*P* value
Induction therapy with alkylators	1.70 (1.16–2.50)	**0.006**
Radiation therapy	1.54 (1.03–2.30)	**0.037**
CAR-T Cell Therapy	1.75 (0.88–3.48)	0.11
Lenalidomide maintenance Post auto-SCT	2.93 (1.87–4.59)	**<0.001**
CR after auto-SCT	1.94 (1.33–2.82)	**<0.001**
Year of auto-SCT (reference: 1990–2006)	0.72 (0.44–1.18)	0.2

Bold values identify statistical significance (*p* < 0.05).

### Characteristics and treatment of patients with t-MN

A total of 98 patients developed t-MN (t-MDS = 65, t-AML = 13, t-MDS transformed into AML = 18, CML = 1 and MDS/MPN overlap = 1). The median age at diagnosis of t-MN was 69 (range, 43–85) years and the median time from auto-SCT to diagnosis of t-MN was 59 (7–257) months. Nine (9%) patients had prior CAR-T cell therapy and the median time from CAR-T cell therapy to diagnosis of t-MDS/AML was 11(range, 2–27) months. There were no cases of CAR-T associated T cell malignancy in our cohort. We observed that 60 of 98 (61.2%) patients with t-MDS/t-AML had a loss of all or part of chromosome 5 or 7 or both, 18 (18%) had normal karyotype, 5 (5%) had trisomy 8 and 5 (5%) had other cytogenetic abnormalities. Sixteen patients (16%) were noted to have loss of 17 p and among them 10 had chromosome 5 and/or 7 abnormalities. NGS data is summarized in Table 3. Among t-MN related to CAR-T cell therapy (*n* = 9), 6 (66%) had *TP 53* mutations. In the t-MDS cohort with available NGS (*n* = 46), 13 had high risk and 30 patients had very high risk disease per the IPSS-M risk stratification.

**Table 3:** Characteristics of patients with therapy related myeloid neoplasm (t-MN) following auto-SCT for multiple myeloma.

Variables	Total (*n* = 98) *n* (%)	Allogenic stem cell transplant		
		Yes (*n* = 11) *n*(%)	No (*n* = 87) *n*(%)	*P* value
Age in years at diagnosis of t-MN, median (range)	69 (43–85)	61 (51–73)	70 (43–85)	**0.01**
Male	62 (63%)	8 (73%)	54 (62%)	0.74
Median time from auto-SCT to t-MN (months), median (range)	59 (7–257)	47 (7–257)	61 (12–236)	0.22
Classification of t -MN–no (%)				0.45
MDS	65(66%)	6 (55%)	59 (68%)	
Therapy related AML	13(13%)	1 (9%)	12 (14%)	
MDS transformed into AML	18(18%)	4 (36%)	14 (16%)	
MPN	1 (1%)	0	1 (1%)	
MDS/MPN overlap	1 (1%)	0	1 (1%)	
Karyotype				0.32
Complex	50 (51%)	6 (56%)	44 (51%)	
Monosomal	22(22%)	1 (9%)	21 (24%)	
Normal	18(18%)	4 (36%)	14 (16%)	
NA	8 (8.1%)	0	8 (9%)	
IPSS-R risk (*n* = 83)				0.26
Very low	0	0	0	
Low	10 (12%)	0	10 (14%)	
Intermediate	14 (17%)	1 (10%)	13 (18%)	
High	22 (27%)	3 (30%)	19 (26%)	
Very high	26 (31%)	6 (60%)	20 (27%)	
NA	11 (13%)	0	11 (15%)	
ELN 2022 classification (*n* = 31)				0.39
Favorable	1 (3%)	0	1 (4%)	
Intermediate	4 (13%)	2 (40%)	2 (8%)	
Adverse	25 (81%)	3 (60%)	22 (85%)	
NA	1 (3%)	0	1 (4%)	
NGS analysis				
*TET2*	5 (5%)	1 (9%)	4 (5%)	0.46
*DNMT3A*	10 (10%)	2 (18%)	8 (9%)	0.31
*ASXL1*	6 (6%)	2 (18%)	4 (5%)	0.13
*RAS pathway*	2 (2%)	0	2 (2%)	>0.99
*RUNX1*	5 (5%)	1 (9%)	4 (5%)	0.46
*Tp53*	39 (40%)	2 (18%)	37 (42%)	0.19
*EZH2*	1 (1%)	0	1 (1%)	>0.99
*SRSF2*	3 (3.0%)	1 (9%)	2 (2%)	0.30
Cytarabine based dose intense chemotherapy	10 (11%)	3 (27%)	7 (8%)	**0.02**
Hypomethylating agent with/without venetoclax	45 (44%)	8 (73%)	37 (43%)	
Best supportive care	35 (37%)	0	35 (40%)	
Lenalidomide	1 (1%)	0	1 (1%)	
Tyrosine kinase inhibitor	1 (1%)	0	1 (1%)	
NA	6 (6%)	0	6 (7%)	
Alive at last follow up	8 (8%)	5 (45%)	3 (3%)	**<0.001**

*NA* Not available.

Bold values identify statistical significance (*p* < 0.05).

Among patients with t-MN, 10 (11%) received cytarabine based dose intensive chemotherapy (7 + 3 regimen) whereas 45 patients (44%) received hypomethylating agents with or without venetoclax (Table 3). Eleven patients (11%) received an allo-SCT (t-MDS = 6, t-AML = 1, t-MDS transformed to AML = 4) at a median time of 6.5 months (range, 3–29) months from diagnosis of t-MDS/AML. Nine patients (82%) received a reduced intensity conditioning (RIC) [fludarabine and melphalan (Flu + Mel) = 5, Flu + Mel, TBI = 3, busulfan and fludarabine (BU-FLU) = 1] and 2 patients received myeloablative conditioning (BU-FLU = 1, BU-FLU and thiotepa = 1) followed by matched unrelated donors (MUD) (*n* = 8), matched related donor (MRD) (*n* = 2) or haploidentical transplant (*n* = 1).

**Fig. 3:**
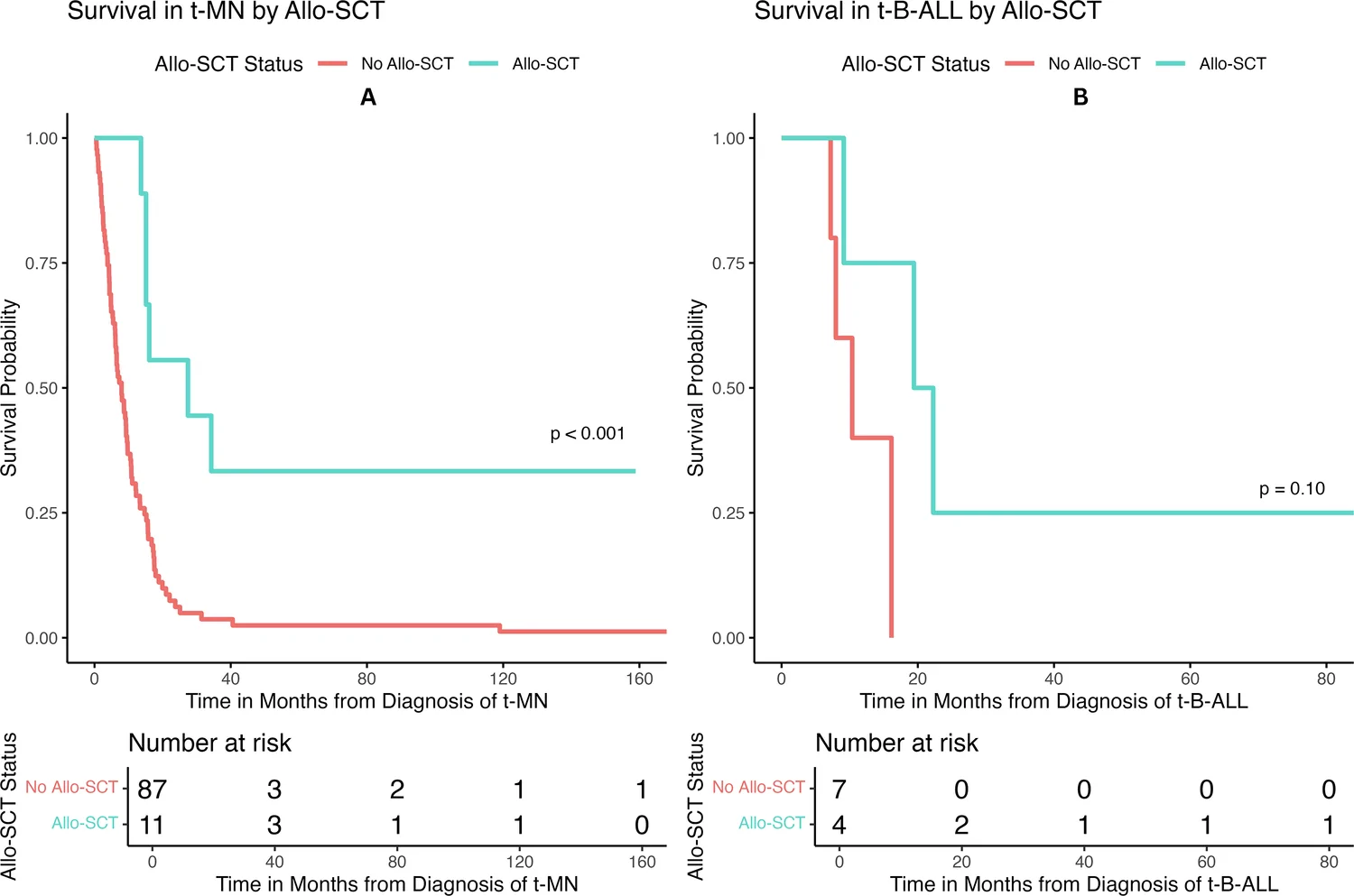
Overall survival by allogenic stem cell transplantation. **A** Overall survival in t-MN by allogenic stem cell transplantation: median 27.4 months vs 7.9 months; hazard ratio 0.25, 95% C.I, 0.15–0.41; *P* < 0.001. **B** Overall survival in t-B-ALL patients by allogenic stem cell transplantation: median 20.9 months vs 10.2 months; hazard ratio 0.35, 95% C.I, 0.07–1.72; *P* = 0.10.

At the time of analysis, at a median follow up of 156.7 months (range, 0.5 to –169.5 months) from diagnosis of t-MN, 8 patients are alive including 5 who underwent an allo-SCT. Among t-MDS/AML patients, the median OS was 8.3 (95% C.I, 6.5–9.7) months from the diagnosis of SHM. The median OS among allo-SCT recipients versus non-recipients was 27.4 (95% C.I, 13.4 to not reached) months vs 7.9 (95% C.I, 5.9–9.4) months, *P* < 0.001 (Fig. 3A). The median follow up among the 11 allo-SCT recipients was 52.2 (range, 0.1–153.4) months and the median OS was 9.8 (4.5 to not reached) months from allo-SCT. Among the 6 allo-SCT recipients that died, 3 had post-transplant relapse of their t-MDS/AML and 3 died from transplant related complications (NRM -27%).

### Characteristics and treatment of patients with therapy related B-ALL (t-B-ALL) and mixed phenotype acute leukemia (MPAL)

Eleven patients developed t-B-ALL and one patient developed MPAL, and their clinical characteristics are summarized in Table 4. The median time from auto-SCT to diagnosis of t-B-ALL was 62.5 (range, 35–127) months. Nine patients received lenalidomide maintenance post auto-SCT and the median duration of lenalidomide maintenance was 28 (range, 6–57) months. Six patients were receiving lenalidomide maintenance at the time of their t-B-ALL diagnosis. Among patients with available cytogenetics, deletion of chromosomes 5 and/or 7 were seen in two patients. Hypodiploidy was observed in four patients and among them three had low hypodiploidy. One patient had a Philadelphia-like phenotype. FISH of bone marrow aspirates of 3 patients with t-B-ALL who were on lenalidomide maintenance revealed an *IKFZ1* deletion and one patient had 17 p deletion. Among 2 patients with available next NGS data, one had *TP53* mutation. The most commonly used induction regimen was Hyper-CVAD based chemotherapy. Four patients with t-B-ALL and one patient with MPAL underwent an allo-SCT. All 5 patients received RIC [Flu + Mel, TBI = 3; fludarabine and TBI = 1; BU-FLU, anti-thymocyte globulin and TBI = 1)] followed by MRD (*n* = 3) and MUD (*n* = 2) allo-SCT. At a median follow up 83.6 (range, 0.7–83.6) months, the median OS from diagnosis of t-B-ALL patients was 15.9 months (95% C.I, 7.1–21.9) for the entire cohort and was 20.9 (95% C.I, 9.0 to not reached) and 10.4 (95% C.I, 7.1 to not reached) months for allo-SCT recipients and non-recipients respectively (*P* = 0.10) (Fig. 3B). The patient with MPAL remains in CR after allo-SCT at a follow-up of 32 months. Three patients died from transplant related complications.

**Table 4:** Characteristics and outcomes of therapy related B- ALL and mixed phenotype acute leukemia patients.

Variables	B-cell ALL (n = 11)	Mixed phenotype acute leukemia (n = 1)
Age at diagnosis, median (range) years	70 (46–77)	66
Male	6 (54.5%)	0
Patients who received post ASCT lenalidomide maintenance	9 (82%)	1 (100%)
Median duration of lenalidomide maintenance prior to diagnosis of B-ALL, median (range) months	28 (6–57)	51
Cytogenetic group		
t (9;22) BCR/ABL1 negative	10	
Hypodiploidy	4	
Low hypodiploidy	3	
Hyper diploidy	1	
Normal Karyotype	2	1
Complex karyotype	4	
BCR-ABL1-like ALL (P2RY8/CRLF2 fusion)	1	
IKZF1 deletion	3	
17 P deletion	1	
TP 53 mutation	1	
NA	2	
Induction chemotherapy		
Hyper CVAD	4	
Mini hyper CVD +/- Inotuzumab	6	1
E 1910 protocol	1	
Complete response /complete response with incomplete count recovery	9	1
NA	2	
Allo-SCT	4 (36%)	1 (100%)
Donor type		
Matched related	2 (50%)	1 (100%)
Matched unrelated	2 (50%)	
Haploidentical	0	
Conditioning regimen		
MAC	0	
RIC/NMA	4 (100%)	1(100%)
Alive at last follow up	4 (36%)	1 (100%)

*RIC* reduced intensity conditioning, *NMA* non myeloablative, *MAC* myeloablative conditioning, *NA* not available.

### Predictors of OS in patients who developed SHM

On univariate analysis, factors predicting better OS after development of SHM were receiving an immunomodulatory drug as induction and receiving an allo-SCT. In multivariate analysis, these factors remained significantly associated with OS (Table 5). In our cohort, SHM (*n* = 85) was the leading cause of death among patients with MM who developed SHM.

**Table 5:** Univariate and multivariate regression analysis of association between patient characteristics and overall survival for patients who developed SHM: Cox regression model.

Variables	Univariate		Multivariate	
	Odds ratio (95% CI)	*P* value	HR (95% CI)	*P* value
Age ≥ 60 at SHM diagnosis	1.23 (0.72–2.1)	0.45	Not included	
male	1.21 (0.80–1.84)	0.36	Not included	
Induction therapy with alkylating agents	1.48 (0.99–2.23)	0.06	Not included	
Induction therapy-IMIDs	0.57 (0.38–0.85)	**0.006**	0.59 (0.37–0.96)	**0.03**
Induction therapy with proteosome inhibitors	0.90 (0.59–1.37)	0.64	Not included	
Lenalidomide maintenance post auto-SCT	0.65 (0.42–0.99)	0.05	Not included	
Complex karyotype (yes vs no)	1.21 (0.77–1.88)	0.41	Not included	
TP53-mutant vs wild type	1.50 (0.98–2.29)	0.06	1.56 (0.95–2.55)	0.08
ASXL1 mutant vs wild type	0.51 (0.16–1.62)	0.26	Not included	
DNMT3A mutant vs wild type	0.75 (0.37–1.5)	0.42	Not included	
RUNX1 mutant vs wild type	0.65 (0.24–1.77)	0.40	Not included	
TET2 mutant vs wild type	0.44 (0.16–1.21)	0.11	0.50 (0.18–1.37)	0.20
IPSS-R higher risk (yes vs no)	1.11 (0.66–1.86)	0.69	Not included	
Allo-SCT (yes vs no)	0.23 (0.12–0.47)	**<0.001**	0.35 (0.14–0.84)	**0.018**

Bold values identify statistical significance (*p* < 0.05).

## Discussion

The treatment paradigm for MM continues to evolve with improvements in OS in the era of novel immune and cellular immunotherapy. As patients are living longer, the risk of delayed complications including SHM is becoming more obvious. Most of the SHM (65%) in our cohort occurred after the first 50 months, reiterating the fact that long-term follow-up is necessary to capture these late events. Our study is one of the largest single center studies with extended follow-up and it aligns with previous studies looking into the incidence of SHM in patients with MM[16, 17]. We observed that receiving an alkylator based induction therapy, radiation therapy, achieving a CR after auto-SCT, use of cyclophosphamide for stem cell mobilization and lenalidomide maintenance therapy was associated with a higher risk of developing a SHM. It is important to highlight that the incidence of SHM is increasing in the novel agent era.

SHM in patients with MM is mediated by disease related, therapy related and patient specific factors[18]. T-MN is the most common SHM post auto-SCT with a cumulative incidence of 1–4% and it tends to have aggressive biology with high-risk features[11, 19–21]. We observed the same in our cohort with most patients having complex karyotypes and *TP 53* mutation. Prior studies have shown that older age and male gender is associated with increased risk of t-MN[17, 22]; however, this was not observed in our study. Therapy related factors also play an important role and the association between exposure to alkylating agents and risk of t-MN is well established[8, 23]. It has been debated whether it is the myeloablative high dose melphalan used in conjunction with auto-SCT or is it the alkylators given as induction therapy that increases the risk of t-MN and prior studies have shown that it is the latter which is the likely cause of t-MN[24]. We observed a similar finding as receiving alkylator based induction therapy emerged as an independent predictor of developing SHM. Fortunately, this is less of a concern in the contemporary quadruplet induction era where alkylators are rarely used. Further, we noted that high dose cyclophosphamide for stem cell mobilization also increases the risk of SHM; however, it is rarely used nowadays given the successful mobilization and less toxicity with granulocyte colony-stimulating factor and plerixafor. The role of radiation therapy in promoting SHM in MM patients is controversial as we lack data from high quality studies. The US connect MM registry study did not find any association between radiotherapy and secondary malignancies[25]; however, the role of radiation therapy in causing SHM in cancer survivors is well established[26, 27] and it is logical to conclude that a similar risk could be applied to patients with MM. In our study, 24% of auto-SCT recipients received radiation therapy and it emerged as an independent predictor of developing SHM. We observed that patients who achieved a CR after auto-SCT had a higher likelihood of developing SHM, and this is likely due to the greater risk of developing complications in long-term survivors.

In addition to the risk of t-MN, patients with MM are also at risk for t-B-ALL which is a rare and distinct SHM in the era of lenalidomide maintenance therapy. A safety meta-analysis of randomized controlled trials highlighted the increased incidence of t-B-ALL in patients with MM who received lenalidomide[28]. Prior studies from Barnell et al. had shown that both t-B-ALL and MM arise from different clones and not from clonal dedifferentiation of MM[29]. Lenalidomide is known to promote leukemogenesis by inducing mutations or deletions in *IKZF1* leading to degradation of Ikaros resulting in maturation arrest of mutated B cell progenitors[30]. In addition, Geyer et al had previously reported that t- B-ALL in the setting of lenalidomide maintenance tends to have a high incidence of *TP53* mutations and low hypodiploidy[31]. We observed a similar finding in our cohort. Since lenalidomide is the main driver of leukemogenesis, drug discontinuation would be the first step that may lead to remission in some patients[31].

Although the risk of SHMs after auto-SCT is low, the clinical outcomes are poor, and it remains a life limiting event for the majority of patients. The key question in the era of highly effective quadruplet induction therapy is whether it is necessary for all auto-SCT eligible MM patients to undergo transplant or can we tailor the consolidation strategy based on the MM risk and minimal residual disease (MRD) status to carefully select patients who are likely to benefit the most from auto-SCT and probably deescalate and forego auto-SCT in standard risk MM patients who have achieved a post induction MRD negative status. The DETERMINATION study utilizing triplet (bortezomib, lenalidomide and dexamethasone) induction therapy showed that the 5-year progression free survival (PFS) was similar in patients who achieved a negative MRD status at the start of maintenance therapy regardless of whether they received auto-SCT[32]. Similarly, the MIDAS trial, a phase 3 study utilizing quadruplet induction therapy and MRD adapted consolidation strategy in transplant eligible MM showed no significant difference in the rate of MRD negativity at 10⁻⁶ sensitivity in standard risk MM patients who received auto-SCT vs those who received quadruplet consolidation therapy[33]. However, long term outcomes from the study are not available with respect to PFS. These studies challenge the role of upfront auto-SCT in patients who have already achieved early MRD negative status in the contemporary quadruplet induction era. Since patients who are MRD negative are likely to be long-term survivors, they will be at greater risk for a SHM and it is now reasonable to ask whether standard risk MM patients who achieve MRD negativity after induction therapy should have auto-SCT deferred.

Lenalidomide maintenance therapy post-auto-SCT in MM remains standard of care as it improves PFS and OS [21, 34] ; however, lenalidomide maintenance increases the risk of SHM as well [10, 35]. We observed a similar finding in our cohort as maintenance therapy with lenalidomide emerged as an independent predictor of SHM. Sperling et al had previously shown that lenalidomide induces t-MN through expansion of pre-existing TP53 mutated clones [36]. Given the potential toxicities associated with indefinite lenalidomide maintenance, there are several ongoing trials looking at time limited therapies and MRD based de-escalation of maintenance therapy. The MASTER trial and MRD2 STOP study has shown that MRD based de-escalation is feasible in a select group of MM patients who achieve a sustained MRD negative status [37, 38]. The Greek myeloma study group showed stopping maintenance therapy after 3 years in patients achieving MRD negativity resulted in continued response and a low relapse rate [39].

B cell maturation antigen directed CAR-T cell therapy has revolutionized the therapeutic landscape of MM; however, the risk of t-MN is well established. In the CARTITUDE -1 trial, 10% of patients treated with ciltacabtagene autoleucel developed t-MN [40]. However, the incidence of SHM was low at 1.7% in the real-world study of ciltacabtagene autoleucel [41]. In our cohort, 5% of CAR-T recipients developed t-MN. Another worrisome aspect is the short latency period from CAR-T cell therapy to diagnosis of t-MN (6–10 months) when compared to more than 5 years with alkylating agents[42]. Our data reiterates these findings, and the median latency period was 11 months in our cohort and a significant proportion of them (66%) harbored *TP 53* mutation resulting in poor outcomes. T-MN in CAR-T recipients have been attributed to CHIP given the higher incidence noted in this population (30–50%)[43]; however, a recent work by Gustine et al has shown that CHIP does not increase the risk of t-MN in CAR-T recipients[44]. Hence, other factors such as age, prior exposure to genotoxic therapy could be playing a role. In addition to t-MN, CAR-T cell therapy has been associated with secondary T cell malignancies[45]. Fortunately, no CAR-T associated T cell malignancies have been reported in our cohort. Given the durable remissions seen with CAR T-cell therapies even in the late line setting as highlighted by the long term follow up of CARTITUDE -1 study[46], it is obvious that the benefits clearly outweigh the potential risks. Nevertheless, since CAR-T has moved into the second line setting, it will be important to have shared decision making with the patient fully understanding the risk involved and perhaps reserving CAR-T at first relapse to patients with high risk or functional high-risk disease. Further research focusing on understanding the risk factors for CAR-T associated t-MN is crucial.

CHIP is common in patients with MM and is clonally unrelated to the underlying plasma cell dyscrasia[47]. There is growing interest in better understanding the impact of CHIP on MM outcomes and risk of SHM. The CHIP clone may expand over time under the selection pressure of various treatment modalities and previous studies have shown that pre-existing CHIP may increase the risk for t-MN post-auto-SCT[48–50]. In addition, presence of CHIP at the time of auto-SCT has been associated with poor outcomes in MM patients[51]. The concept of screening for CHIP and monitoring the expanding CHIP clone to identify high risk patients appears intriguing. In a recent work, the Spanish (PETHEMA/GEM) cooperative group reported that screening for CHIP mutations in MM patients is unlikely to be beneficial as the mutant CHIP dynamics are not associated with outcomes[52]. Further studies are needed to ascertain the role of monitoring for evidence of expansion of an existing clone during maintenance therapy that could potentially identify patients at highest risk of developing SHM.

Allo-SCT is a potentially curative therapy in a select group of patients with SHM; however, the majority are ineligible due to age, comorbidities and other factors. Consistent with prior studies, we observed that only 10% of patients with SHM received an allo-SCT. Prior studies have highlighted the role of allo-SCT in improving long term outcomes of patients with t-B-ALL[53, 54]. In our cohort of t-B-ALL, we observed a trend towards better OS in allo-SCT recipients. T-MN tends to be inherently chemotherapy resistant and are often associated with poor survival even with allo-SCT. A recent European Blood and Marrow Transplant registry study looking at outcomes of MM patients with t-MN receiving allo-SCT had shown very high rates of disease relapse (35% at 1 year), NRM (20% at 1 year) and less than one third of the patients achieving durable disease-free survival. Older (>65 years) patients with high-risk cytogenetics did not benefit from allo-SCT[55]. We observed a similar finding in terms of disease relapse and NRM. Although, in our cohort of t-MN patients, median OS was better for those who received an allo-SCT compared to the non-transplant group, this can be explained by the difference in the baseline characteristics as the allo-SCT recipients were younger (*n* = 7 (64%), age < 65), only two patients had *TP 53* mutation and approximately one third had normal karyotype[56] which are favorable prognostic factors.

Previous studies looking into the cause specific mortality in MM patients with SPM have shown that the main driver of mortality is MM and non-cancer related causes[57, 58]. A recent CIBMTR study showed that patients with MM who developed a SHM (*n* = 63; [t-MN = 36]) after auto-SCT tend to have a poor OS and our study supports these findings [11]. Intriguingly, the leading cause of death in the CIBMTR study was MM presumably due to fewer patients having t-MN [11]. In our cohort, the leading cause of death was SHM and these findings align with the MD Anderson experience and in both studies t-MN accounted for the majority of SHM [17].

Major strengths of our study include large sample size, availability of cytogenetics data and extended follow up period. In addition, our study did include patients who received upfront novel agents such as anti-CD38monoclonal antibodies and CAR T recipients, thus reflecting contemporary practice patterns. Our study is limited by its retrospective nature and resultant selection bias. In addition, this study spans over three decades during which the treatment of MM and SHM has evolved considerably, and this introduces bias. Unfortunately, we do not have the CHIP data in our cohort and hence we are unable to determine if the SHM were related to CHIP. Also, data on prior malignancy diagnosis was not available and this may increase the risk of SPM/SHM.

In conclusion, this large single institution study highlights the incidence of SHM in MM patients undergoing auto-SCT in the current era and may be used for shared decision making when counseling patients who are eligible for auto-SCT. Although the incidence of SHM is relatively low, it tends to be a very aggressive disease with poor outcomes. A select group of patients with favorable disease biology may benefit from an allo-SCT. Identifying the risk factors for SHM and risk mitigating strategies are crucial to improving long term outcomes.

## Data Availability

Original data will be shared upon reasonable request, please contact Gertz.Morie@mayo.edu.
